# Cohesive Strength and Structural Stability of the Ni-Based Superalloys

**DOI:** 10.3390/ma15010200

**Published:** 2021-12-28

**Authors:** Igor Razumovskii, Boris Bokstein, Alla Logacheva, Ivan Logachev, Mikhail Razumovsky

**Affiliations:** 1 Joint Stock Company “Kompozit”, Pionerskaya Street 4, 141070 Korolev, Russia; ailogacheva@yandex.ru (A.L.); ivan@logachev.biz (I.L.); razmikhail@gmail.com (M.R.);; 2 Department of Physical Chemistry, National University of Science and Technology (MISiS), Leninsky Prospect 4, 119049 Moscow, Russia; bokst@misis.ru

**Keywords:** Ni-based superalloys, cohesive strength, ab initio calculations, single- and polycrystals, grain boundaries, raft-structure, segregation, mechanical properties, additive objects

## Abstract

The influence of alloying elements on the cohesive strength of metal heat-resistant alloys (HRAs) is analyzed. Special parameters are introduced to characterize the individual contribution of each alloying element. These are the partial molar cohesion energy of the matrix (χ) and the cohesive strength of the grain boundaries (*η*) and can be calculated by computer modeling based on the density functional theory. The calculating results of the parameters χ and *η* in nickel HRAs with mono– and polycrystalline structures alloyed with refractory metals are presented. The calculated data are used to select the chemical composition and develop new nickel (Ni) HRAs with superior creep-rupture properties. It is assumed that a similar approach can be used to search for alloying elements that will contribute to increasing the cohesive strength of additive objects. The resistance of coherent γ-γ′ and lamellar (raft) structures in nickel HRAs to the process of diffusion coarsening during operation is analyzed.

## 1. Introduction

The most important performance characteristics of heat-resistant alloys (HRAs) are creep and fatigue resistance, which are very complex functions of chemical composition and microstructure. The microstructure of metal HRAs, among which the first place is occupied by Ni-based alloys [1,2,3], usually consists of two main phases: a solid solution based on the main element containing alloying elements (matrix), and a strengthening phase, which is usually used as intermetallides, carbides and silicides.

The metal matrix of the HRAs can have both a monocrystalline and polycrystalline structure. The single-crystal structure is usually obtained by directional solidification methods. The most typical example of such monocrystalline objects are the blades of gas turbine engines of aircraft made of nickel HRAs. Single crystals are characterized by higher creep resistance compared to polycrystals. As a result, one of the main last decades successes of HRAs manufacturing technology is the development of the casting process for single-crystal blades of gas turbine engines [4]. The transition from polycrystalline parts to single crystals made it possible to eliminate the weakest element of the structure of polycrystals—high-angle grain boundaries (GBs). Compared to the bulk, GBs have excessive energy, increased diffusion permeability [5,6], and other characteristics which contributes to the acceleration of creep of alloys at elevated temperatures under load.

The main gas turbines blades and disks heat resistance characteristics are fatigue resistance and creep resistance. For gas turbine blades, the most important characteristic is creep resistance, since they operate under alternating loads and, as a rule, they are made of monocrystalline alloys. The disks operate under conditions of simultaneous action of thermocyclic, cyclic loads and tensile loads. For discs, the most important characteristic of heat resistance is fatigue resistance. Ni-based alloys obtained using powder metallurgy technology have a higher fatigue properties due to the dispersed structure provided by this technology. Such alloys are used in aircraft and rocket engines [7,8].

An important factor of high heat resistance of HRAs is the strength of interatomic bonds in the crystal lattices of the phases that make up the structure of the alloys and first—the alloy matrix [9]. The characteristic of the binding forces of atoms in the crystal lattice of solids is the cohesive energy [10]—this is the energy necessary for the decomposition of a crystal into free atoms, and it can be obtained as the difference between the total energies of an atom in the free state E_at_, and a crystal, E_crist_. To increase the interatomic interaction forces, the alloying elements introduced into the alloy should improve the cohesion properties of the alloy. For improvement the performance characteristics of polycrystalline alloys, it is necessary to develop approaches to enhance not only the cohesive energy of the matrix, but also to increase the cohesive strength of the GBs.

In this paper, the theoretical methods for calculating the parameters of the cohesive strength of the HRAs are described and the results of applying the theory to the development of experimental Ni-based HRAs with mono—and polycrystalline structures are presented. The features of the γ-γ′ microstructure Ni-based single crystal superalloys and the kinetics of its diffusion coarsening process, which determines the operational resource of the material, are also considered.

## 2. Theoretical Estimate of the Effect of Alloying on GB Cohesion and the Bulk

For a theoretical alloying elements effect assessment on the cohesive energy *E_coh_* of the alloy matrix (solid solution), it is proposed to use the partial molar cohesive energy *χ_i_* = ∂*E_coh_*/∂*c_i_* [11]. It is assumed that the cohesive energy of the alloy can be represented as a sum of the major (matrix) element cohesive energy (for example, Ni), the corresponding partial contributions from the alloy components and *E_coh_*(0):

(1) $$E_{coh}=E_{coh}\left(0\right)+\underset{i}{\sum }c_{i}\chi_{i}\equiv E_{coh}\left(0\right)+\varepsilon$$

where *ε* is the additional gain of the cohesive energy due to alloying.

The values of *χ_i_* can be calculated from the first principles within the framework of the density functional theory (DFT) [11,12]. Cohesion affects the strength properties, and it is expressed in terms of a physical quantity called the partial molar energy of cohesion, which depends on the influence of alloying elements. The cohesion energy and strength of the alloy increase with a positive value of the parameter χ according to the theory. The higher the value of χ, the more effectively the alloying element should increase the long-term strength.

Rice, Wong and Thompson proposed a theoretical solution [13,14] for estimating the cohesive strength of the interface and they described approach that was centered around evaluation of ideal work grain boundary (GB) separation Wsep [15], that is defined as the reversible work required to cleave GB into two free surfaces (FS). Wsep controls the mechanical properties of the surfaces:

(2) $$W_{sep}=2\gamma_{fs}-\gamma_{gb}$$

where *γ_gb_* and *γ_fs_* are the energies of GB and the respective FS per unit area.

The impurity segregation to the GB changes GB separation work, and GB cohesive strength containing segregated solute can be calculated using the strengthening energy parameter *η* [12]:

(3) $$\eta =(W_{sep}-W_{sep}\;(0))/\Gamma$$

where *W_sep_* and *W_sep_*(0) stand for separation work with and without segregating atoms and *Γ* is the interfacial atoms excess. Parameter *η* can be calculated by DFT directly using the free surface and GB segregation energies of the solute atoms under special condition:

(4) $$\eta =E_{gb}\;-\;E_{fs}$$

where *E_gb_* and *E_fs_* are segregation energies of the impurity corresponds to the free surface and to the GB, respectively.

Ability of the chemical elements to segregate to a GB [16] and determination of their effect on the cohesive strength of GBs are two important criteriums for assessment of chemical elements on the state of GBs in alloys. GBs cohesive strength experimental determination is a complex problem, and it has been the subject of an experimental study rarely.

The experimental data on segregation of impurities to GBs in HRAs are scarce. There are two main groups of experimental methods for studying the segregation of impurities on GB.

The first group of methods (atom probe tomography (APT) and Auger electron spectroscopy (AES)) allows one to directly measure the concentration of the segregant on the GB. Both methods have significant limitations in the choice of research objects. AES is successfully used in cases where there is a brittle destruction of the polycrystal along the GB. In the APT method, the objects of study are needle-shaped samples that need to be prepared from massive objects. These circumstances greatly narrow the range of objects of direct experimental studies of the chemical composition of GBs.

Another method is known that allows us to indirectly find the value of the GB segregation coefficient s in a diffusion experiment via measuring the diffusion penetration profiles of the given element along GB in B mode and C mode according to the Harisson classification [17].

An effective approach to the theoretical study of the elements segregation to GB and the cohesive GB’s strength are ab-initio calculations within the DFT’s framework [18].

The best way to study the components segregation at the solids interfaces are experimental and theoretical studies combinations. The following are some combined studies examples of the elements segregation on GB in metals alloys includes Ni-based HRAs.

Microchemistry of GB in Ni-based HRA Astroloy, that alloying system includes Ni, cobalt (Co), chrome (Cr), molybdenum (Mo), aluminum (Al), titanium (Ti), zirconium (Zr), boron (B) and carbon (C), have been investigated by Blavette et al. [19]. The theory predictions are confirmed by the results of atom probe tomography revealed the presence of Mo, Cr, B at GBs in [12].

Ab-initio study of GB segregation in nickel-base alloys and a combined atom probe tomography were conducted in [20]. The investigation methods comprise DFT calculations and APT measurements. Alloying elements segregation to GBs of 725 Ni-base alloy in three modifications was investigated. The experimental concentration profiles analysis across the GBs has revealed strong boron’s segregation and pronounced molybdenum’s segregation. Weak nonmetals (C, oxygen (O), phosphorus (P) and silicon (Si)) segregation was detected at the GBs. DFT-based segregation model has supported the APT analysis data. Mo and B of all studied solutes show the most pronounced enrichment tendencies at the GB. These elements have the potential to replace other undesirable solutes from the interface consequently.

The diffusion experiment is also an effective tool for the experimental determination of the grain boundary segregation coefficient [21,22]. A detailed parameters comparison of the elements segregation into the GB determined via the diffusion experiment and ab-initio calculation in DFT framework is carried out in [22] that investigated the several elements (silver (Ag), gold (Au), selenium (Se), germanium (Ge), Ni, Co, and bismuth (Bi)) segregation characteristics into GB in copper (Cu). These results are illustrated that Cu bicrystals for a special GB Σ5 (210) [100] and diffusion for DFT calculations a well correlation is shown between the experimental and theoretical data in the case of model objects.

Taking into account the observed good correlation between the results of experimental and theoretical studies of the segregation of alloying elements on GB, calculated data can be used, if they are easier to obtain, to modify the chemical composition of HRAs in order to increase the cohesive strength of GB and increase creep resistance.

Thus *E_gb_*, *η*, and *χ_i_* paraments combination provides one with a powerful composition HRAs design tool. In terms of cohesive strength, the most “useful” alloying elements should increase the cohesive strength of the matrix phase (that is, increase the *E_coh_*) and the GB (increase *η*) during alloying simultaneously. Due to the segregation of alloying elements to the GB, useful additives can be introduced into the alloy in a small amount (the concept of low-alloying additions [12]).

## 3. The Design of Ni-Based Single Crystal Superalloys

**Figure 1:**
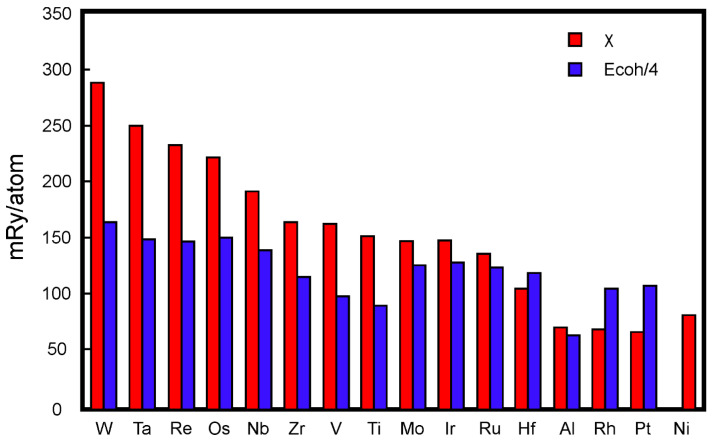
Calculated values of the χ parameter and the experimental cohesive energies (divided by 4) of alloying elements in Ni-based alloys [10,11]. Copyright 2008 Elsevier.

The results of the calculation of the parameter *χ* for several alloying elements (Ti, Al, Zr, hafnium (Hf), vanadium (V), niobium (Nb), tantalum (Ta), Mo, tungsten (W), rhenium (Re), ruthenium (Ru), osmium (Os), rhodium (Rh), iridium (Ir), and platinum (Pt) in Ni-based alloys are shown in Figure 1 [11]. Figure 1 first shows that the most efficient alloying additions to the Ni-based superalloys are W, Ta and Re, and secondly indicates the primary role of W in ensuring high cohesive strength of the nickel HRAs.

It is interesting to note in this regard that such well-known Ni-based HRAs as MAR-M200 and GS6U developed many years ago did not contain Ta and Re as alloying additives at all but included a relatively large amount of W (concentrations are indicated weight percentages (wt. %) in further): 12.5 in the alloy MAR-M200 and 10.5 in the alloy GS6U. Later, when first Ta and then Re were included in the HRAs alloying system, W was partially replaced by Ta and Re. As a result, W amount in the HRAs gradually decreased with an increase in Ta and Re concentration. For instance, the tendency can be easily seen in several CMSX superalloys (CMSX is a trademark of the Cannon-Muskegon Corporation) chemical compositions evolution. W concentration in the CMSX-2 (the first Ni-base generation superalloys for single crystal blades) was 8 and Ta concentration—6. In CMSX-4 superalloy (the second generation) W concentration was 6, also it consisted of 6.5 Ta and 3 Re. Finally, Ta and Re concentration was 8 and 6, respectively, and W concentration was reduced to 5 in the CMSX-10M superalloy (the third generation).

Although Ta and Re have large and approximately equal values of the parameter *χ*, and therefore can equally strongly increase the creep resistance of the alloy, it is interesting to introduce Ta and Re into the HRAs not only at the expense of W, but at the expense of Ni while maintaining a high concentration of W. If we consider the high solubility of W in Ni, this approach seems quite reasonable.

Several similar alloys KS-(1-3) with an increased W content were proposed in [11,23]; the chemical compositions of KS alloys are shown in Table 1.

**Figure 2:**
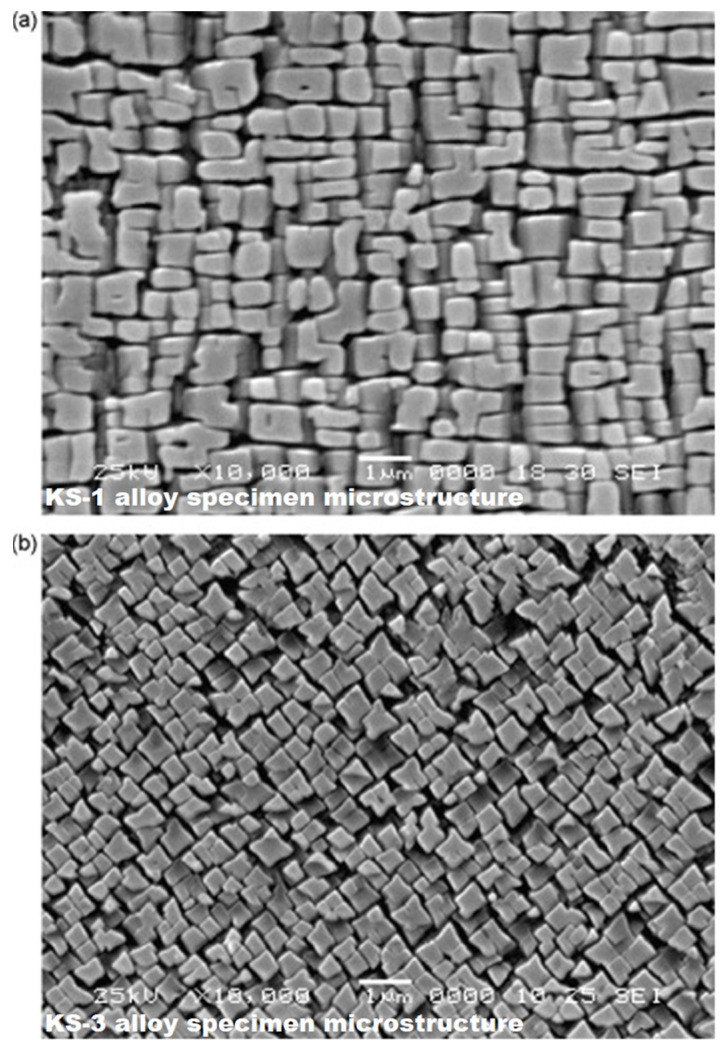
Coherent γ-γ′ microstructures of the KS-1 (**a**) and KS-3 (**b**) alloys specimens were producing via complete heat treatment included hot isostatic pressing (HIP), homogenization and two-step aging. Copyright 2008 Elsevier.

KS alloys with a single-crystal structure after complete heat treatment had a coherent γ-γ′ microstructure typical for nickel HRAs, Figure 2. In the microstructure of cylindrical castings with a growth axis of [100], a typical casting porosity was observed, which was minimized by hot isostatic pressing according to a special regime [24].

The results of long-term tests of the mechanical properties of samples of KS-(1-3) alloys at a temperature of 1000 °C after complete heat treatment are shown in Table 2. Similar characteristics of Ni-based superalloys CMSX for single crystal blades and Ru-bearing superalloy EPM-102 are also presented there for comparison.

Table 2 shows that creep rupture characteristics of KS-1 alloy (5 of Ta) has improved compared with the CMSX-2 (6 of Ta) for σ_100_^1000^ as well as for σ_500_^1000^. Although σ_100_^1000^ value is the same in KS-2 and CMSX-4 alloys, and KS-2 alloy contains less expensive Re amount. According to the table one can see that KS-3 alloy has been improved creep-rupture characteristics compared with CMSX-10M. Comparing the KS-3 alloy with Ru-bearing EPM-102 alloy, which has the highest creep-rupture properties. In addition, one can see that these alloys have the same σ_100_^1000^ (330 MPA) value, but KS-3 again has improved σ_500_^1000^ characteristic.

Using this approach together with other methods of design of Ni-based HRAs, it was developed a series of experimental HRAs with high heat resistance [25].

## 4. Features of the Microstructure of Ni-Based Superalloys and the Kinetics of Its Diffusion Coarsening Process

The microstructure of Ni-based HRAs consists of a γ-matrix (a solid solution of alloying elements in Ni with a face-centred cubic (fcc) lattice) and a strengthening γ′—phase, which is an ordered Ni_3_(Al,Ti)—based intermetallic phase with L1_2_ structure. The intermetallic γ′-phase in Ni-based HRAs is formed because of the decomposition of a supersaturated γ-solid solution during cooling. The result is a coherent γ-γ′ microstructure in which isolated dispersed particles of the γ′ phase are arranged in a regular manner in a continuous γ-matrix, Figure 2.

The coherent (γ-γ′)—microstructure of Ni-based HRAs characterized by a high resistance to the process of its diffusion coarsening at elevated temperatures due to the unique and favorable combination of thermodynamic and kinetic characteristics. Effective dispersion hardening of the HRAs is provided by optimal parameters (γ-γ′) microstructure, among which the most important are the particle size, their number and morphology. To predict the stability of (γ-γ′)—microstructure and the long-term strength of Ni-based HRAs, it is necessary to know the mechanisms of diffusion coarsening of the microstructure and the kinetic parameters of the process.

For the quantitative description processes diffusion-controlled growth of precipitations Avrami equation [26] is used, as a rule. This equation is a generalization of the theoretical approach to the kinetics of the phase transformations:

(5) $$\xi =1-exp\left(-At^{n}\right);\;A=\beta \frac{\Delta G}{kT}exp\left(-\frac{\Delta E_{\varphi }}{kT}\right)$$

where ξ is relative mass of a new phase, ΔG and $\Delta E_{\varphi }$—Gibbs and activation energies; β is constant and n is a parameter which depends on individual properties of growing phases.

The first theory describing the evolution of the size of individual particles of strengthening phases was developed by Wert [27] and Zener [28] and much more fully by Ham [29]. This model is valid at the first stage of growth, with significant supersaturation of the solid solution, when the diffusion transfer occurs under the influence of a concentration gradient.

**Figure 3:**
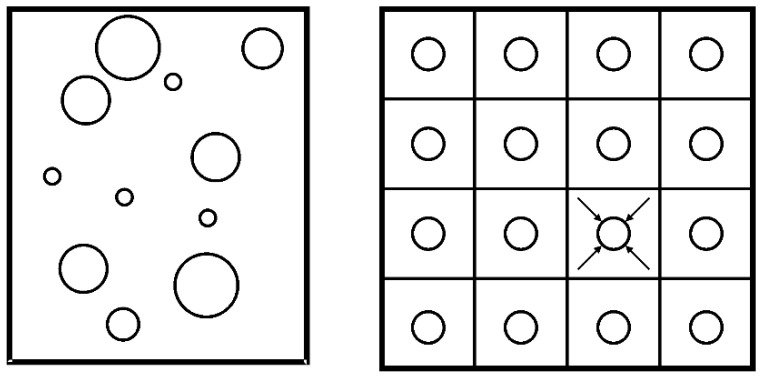
Ham model [29].

In a simple model (Figure 3) proposed by Ham for growth of Fe_3_C particles in Fe, the spherical particles of Fe_3_C grow in solid solution independently one from other.

Balance equation is the same for every particle:

(6) $$\left(c_{2}-c_{1}\right)\frac{dr}{dt}=-D\frac{dc}{dr}=-D\frac{\left(c_{1}-c\right)}{r}$$

In this variant, the size of the growing particle is equal to:

(7) $$r\left(t\right)=r_{0}+\sqrt{2Dt}$$

In this formula, r(t) and $r_{0}$ are the particle size at the time t and at the initial time, respectively, D is diffusion coefficient.

We can describe the growth of the particles in solid solutions in the state close to equilibrium, at the last stage of the growth. Consequently, concentration gradient and diffusion flow are very small. Now we can point out the classic works by Lifshitz and Slyozov, and Wagner (L-S-W) [30,31].

For the solutions close to equilibrium the curvature of the particles surface plays the main role. Concentration of solute in saturated solution near the flat surface $c_{\infty }$ less than near surface of particle with radius *r*—*c_r_*:

(8) $$c_{r}=c_{\infty }exp\left(\frac{2\sigma \Omega }{rRT}\right)$$

where σ—surface tension; Ω—molar volume.

This equation is written by analogy with Gibbs-Tomson equation for vapor pressure. The chemical potential of the component near the surface of the spherical particle with a small radius *r* is:

(9) $$\mu_{i}^{r}=\mu_{i}^{\infty }+RTln\frac{c_{i}^{r}}{c_{i}^{\infty }}$$

where $\mu_{i}^{r}$, $\mu_{i}^{\infty }$, $c_{i}^{r}$ and $c_{i}^{\infty }$ are chemical potentials and concentrations of component near curved and plane surfaces. If in Equation (8) 2σΩ ≪ *rRT*, then

(10) $$c_{r}\approx c_{\infty }\left(1+\frac{2\sigma \Omega }{rRT}\right)=c_{\infty }+\frac{\gamma }{r};\;\gamma =\frac{2\sigma \Omega c_{\infty }}{RT}$$

**Figure 4:**
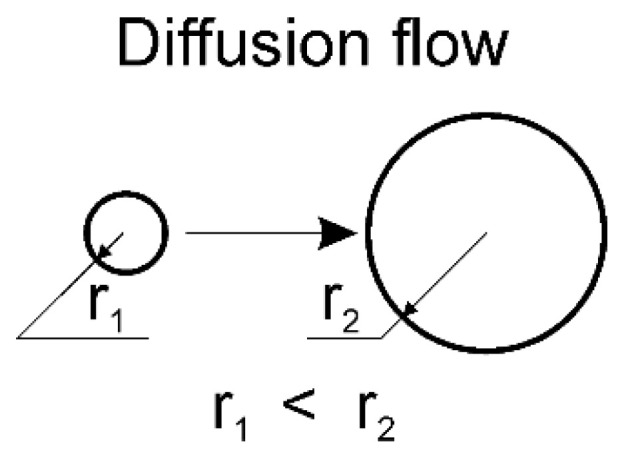
Diffusion flow according to L-S-W theory [30].

Diffusion flow is directed from the particle with small radius to the particle with a big radius (Figure 4). As a result, the big particles grow and the small—dissolute.

Diffusion flow is equal to

(11) $$j=-D\frac{\partial c}{\partial r}=-D\frac{c-c_{r}}{r}=\frac{D}{r}\left(\Delta -\frac{\gamma }{r}\right)$$

where *Δ*—supersaturation *Δ* = *c* − *c*_∞_ ≪ *Δ*_0_.

Balance equation is

(12) $$\frac{\partial r}{\partial t}=\frac{D}{r}\left(\Delta -\frac{\gamma }{r}\right)$$

**Figure 5:**
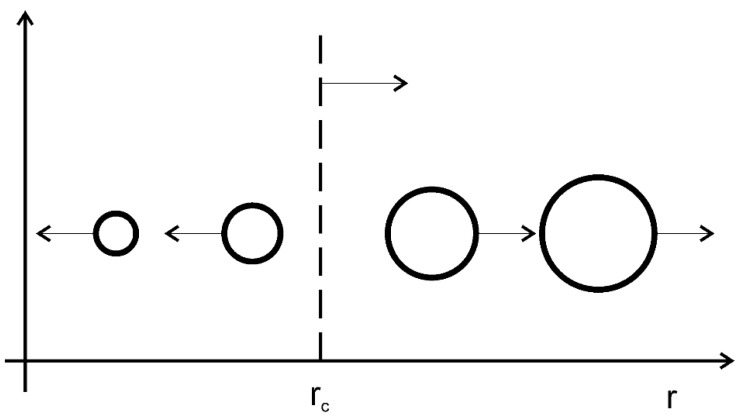
Scheme of diffusion coalescence. Big particles grow, small particles dissolute.

Hence the particles grow ∂*r*/∂*t > 0*, if Δ > γ/*r* or *r* > γ/Δ and vice versa. So, there is a critical radius *r_cr_* such that the particles with radius more than critical—grow and less—dissolute (Figure 5).

Supersaturation decreases and critical radius grows with time. Consequently, the number of particles decrease. According to L-S-W theory

(13) $$r^{3}=r_{0}^{3}+\frac{4}{9}D\gamma t$$

The L-S-W-theory was confirmed in many studies of precipitations growth in conditions of low supersaturation. The parameters of the heterophase microstructure in dispersion-hardening alloys can be changed by heat treatment. In the works of [32,33], it was investigated the influence of the particle size of the γ′- phase on the mechanical properties of the powder Ni—based superalloy EP741NP. It was shown, firstly, that after different heat treatments the experimental histograms of the distribution of γ′- phase particles by the size corresponded to the representations of the L-S-W-theory and secondly, it is possible to vary the particle sizes from 0.5 to 0.2 microns. The difference in the sizes of the γ′-particles of the alloy has a significant influence on the performance characteristics of the P/M EP741NP alloy. Number of cycles to failure *N_f_* at 650 °C increases from *N_f_* = 45,200 (large particles) to *N_f_* = 82,000 (small particles).

Information on various aspects of diffusion phenomena in metal alloys can be found in [34,35].

**Figure 6:**
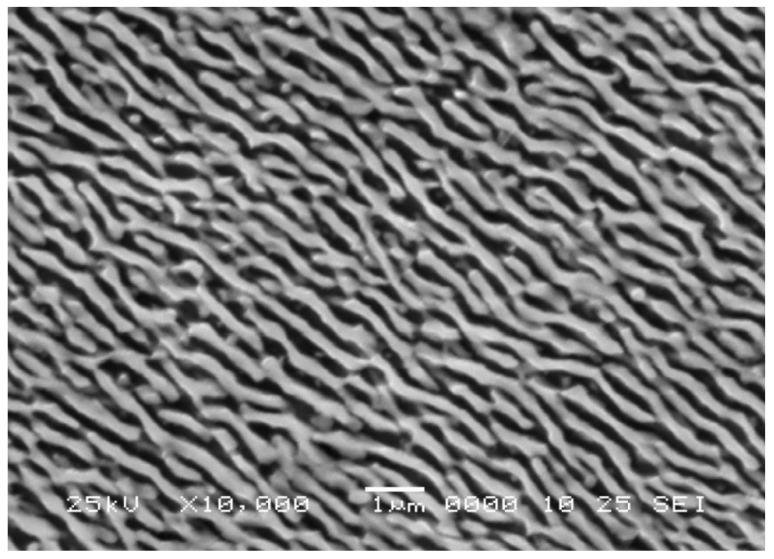
Scanning electron micrograph of longitudinal section of Ni-based single crystal alloy (KS-3, see Section 3) creep tested at 1000 °C and 170 MPa for 100 h [11]. Copyright 2008 Elsevier.

However, during the operation (or when testing mechanical properties) of Ni-based single crystal superalloys at elevated temperatures, isolated γ′—phase particles quickly combine into plates (raft structure). A typical raft structure formed in Ni-based single crystal (KS-3, see Section 3) during creep testing is shown in Figure 6 [11].

The thermodynamic driving force for formation of the raft structure in Ni-based single crystal superalloys is determined by the competition between elastic interactions and surface tension. The elastic interactions mainly depend on the difference between lattice parameters of γ- and γ′-phases (so called misfit), the different elastic moduli of the phases, and the effects of external stress fields [36,37,38]. The time of formation of the raft structure is small compared with the time to rupture. Therefore, it is the stability of the raft structure in relation to the process of its diffusion coarsening that determines the service life of superalloy single crystals during long-term operation.

**Figure 7:**
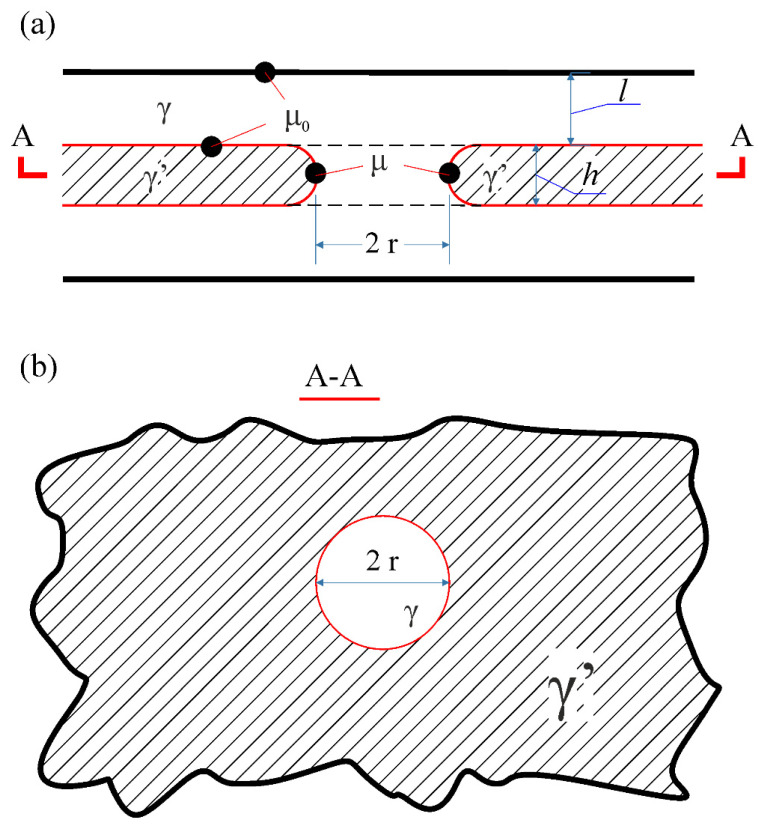
Schematic representation of a hole-like defect in the plate of the γ′—phase; (**a**,**b**)—the sections perpendicular (**a**) and parallel (**b**) to the layers, accordingly [39,40]. Copyright 1994 Elsevier.

The model of diffusion coarsening of lamellar structure consisting of two phases (for example, γ and γ′) is proposed in [39,40,41]. This model is based on the L-S-W theory and assumes that the plates of the γ and γ′—phases have specific defects, namely, the holes which are filled with the material of the adjacent phase, Figure 7.

The formation of a single hole with a radius *r* in γ′—lamellae cause a change in the surface energy by

(14) $$\Delta F_{s}\approx 2\pi \beta hr-2\pi \beta r^{2}$$

where *h* is the initial thickness of the γ′—lamella, *β* is the surface tension.

It follows from expression (14) that there is a critical pore radius *r_c_*, which can be found from the condition ∂F/∂r = 0. To minimize the surface energy, the pores of a small size *r* < *r_c_* should disappear, and the pores with a radius *r* > *r_c_* should unlimitedly grow.

The raft structure coagulation occurs via the diffusion-limited those holes growth which have a size larger than a certain critical value in this case. The diffusion mass transport driving force during the raft structure changes is determined via the difference between atoms chemical potentials near the curved holes edges μ and near a planar interphase $\mu_{0}$, Figure 7. This difference is given by

(15) $$\mu -\mu_{0}=\beta \Omega \left(\frac{2}{h_{\gamma^{\prime }}}-\frac{1}{r}\right)$$

where Ω is the atomic volume. In this formula the holes are considered as a concave surface in the section parallel to a convex surface in the perpendicular section and the layers. The difference μ − $\mu_{0}$ provides diffusion flux occurrence.

(16) $$j=\left(\frac{Dc_{\gamma }}{kT}\right)\nabla \mu \;$$

Here ∇μ denotes the gradient of the chemical potential, D is the effective diffusion coefficient, *c_y_* is the diffusing component concentration in γ phase. It is worth noting, D takes into account diffusion mass transport possibility both in the bulk and along the interphase boundaries.

The radius of a hole changes according to with the equation [40]

(17) $$\frac{dr}{dt}=A\left(\frac{2}{h}-\frac{1}{r}\right)$$

where

(18) $$A≅\frac{c_{\gamma }}{c_{\gamma^{\prime }}-c_{\gamma }}\frac{\beta \Omega }{h^{2}}\frac{\left(D_{v}l+D^{\prime }\delta \right)}{RT}\;$$

Here *l* is the γ—layers thickness, *c_γ′_* − *c_γ_* the difference between diffusing component concentrations in γ′ and γ phases, *D_v_* is the bulk diffusion coefficient in the γ phase, *δ* is γ/γ′ interphase boundaries width, *D′* is the diffusion coefficient along these boundaries, Ω being the atomic volume.

The model assumes that the destruction of the raft structure at elevated temperatures occurs due to its damage because of the growth of hole-like defects over time. There is a maximum allowable size *r_r_* of defects, when reaching which the rupture of the object occurs.

An example of a quantitative assessment of the stability parameters of raft structure in a single-crystal HRA with a growth axis of [100] and a chemical composition of Ni-10Co-9Cr-5Al-2Ti-1Nb-2Hf-12W (wt.%) can be found in [40]. Single-crystal samples of the investigated alloy after directional solidification were subjected to homogenization at a temperature of 1300 °C for 3 h and stepwise annealing according to the regime of 1050 °C–10 h + 870 °C–30 h (initial state).

To form the raft structure, the samples were tested for long-term tensile strength under a load of 170 MPa at a temperature of 1000 °C for 0.3 *t_r_*, where *t_r_* is the time to rupture (*t_r_* = 330–360 h for different samples). The formed raft structure had the following geometrical characteristics: *h* = 0.4 µm, *l* = 0.25 µm. The following diffusion characteristics were used to evaluate the kinetic parameter A at a temperature of 1000 °C: *D_γ_* = 1. 7 × 10^−16^ m^2^·s^−1^, *D*′ *δ* = 4. 8 × 10^−22^ m^3^·s^−1^ [42]. The interphase boundary γ/γ′ in Ni-based superalloys is semi-coherent one and characterized the surface energy of about 18 × 10^−2^ J·m^−2^. The calculated data obtained show that the pores with a size of 0.4 microns in the initial raft structure reach a size of about 1 micron during tests of 100 h. This is the scale of structural changes observed when the creep tests of single crystals are carried out before their rupture.

The hole-like model of diffusion coarsening of lamellar structures was used in [39,41] for a detailed study of the kinetics of the process in a two-phase intermetallic alloy Ti-47.5 at. % Al. Lamellar γ/α_2_ structure in alloys based on the Ti-Al system are of interest, firstly, because it characterizes an important class of intermetallic structural alloys. Secondly, it is convenient model object for quantitative metallographic studies due to the high perfection of the lamellar structure.

To experimentally evaluate the changes in the lamellar γ/α_2_ structure at elevated temperatures, the authors [39] annealed samples with a given microstructure at temperatures of 1373–1073 K for 3–12 h and constructed experimental distributions of holes by size. For the theoretical analysis of experimental data and the determination of the kinetic parameters of the diffusion coarsening process, an expression for the hole size distribution function N (*r*, *t*) was obtained and used for processing experimental data. This made it possible to determine the most important kinetic characteristic of the diffusion coarsening process—the activation energy and compare it with the parameters of self-diffusion and other diffusion-controlled processes in titanium aluminides.

## 5. Theoretical Assessment of the Cohesive Strength of Grain Boundaries and Analysis of the Alloying System of Powder Disk HRAs

For the alloying element to strengthen the GB in the alloy matrix, it must firstly enrich the GB because of segregation, and secondly provide an increase in the cohesive strength of the GB, determined by the parameter *η*. The concept of low-alloying additions [12] developed for polycrystalline alloys additionally assumes that the alloying element that strengthens the GB should provide an increase in the cohesive energy of the alloy matrix, determined by the parameter χ.

**Figure 8:**
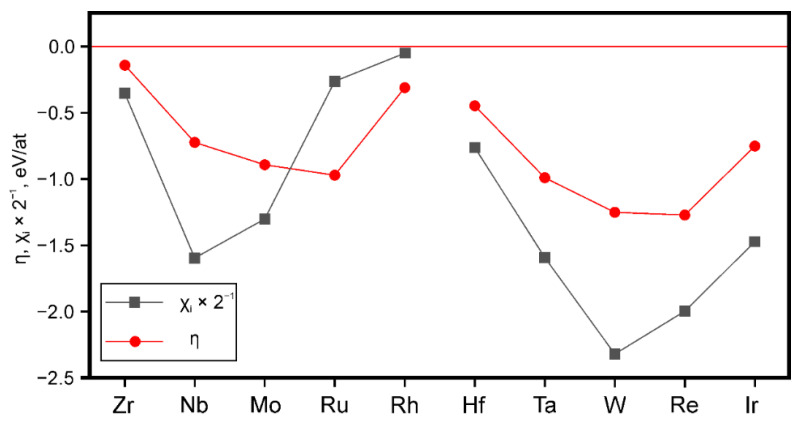
The χ_i_ × 2^−1^ and *η* (eV/at.) parameters values when alloying nickel alloys with refractory transition metals [43]. (Reproduced with permission from Razumovskii IM [43]. Copyright (2020) Pleiades Publishing, Ltd.).

In [12], this approach was used to analyze the chemical composition of the Ni-based HRAs obtained by the powder metallurgy. Figure 8 shows the results of calculating the parameters *χ* and *η* in nickel alloys alloyed with refractory transition metals (Zr, Nb, Mo, Ru, Rh, Hf, Ta, W, Re, and Ir); to study the segregation of elements to the GB, a special GB Σ5 (210) [100] was used in the FCC nickel lattice [12].

The authors [12,43] have established that all the considered refractory metals are characterized via negative E_gb_ and *η* parameters values in nickel alloys; it shown all of them can tend to segregate to GB and tend to increase the cohesive strength of GB. The choice of matrix strengthener is simplified by the correlation in the behavior of the parameters χ and *η* (Figure 8) in Ni-based alloys that is, the GB strengtheners Zr, Hf, Nb, and Ta will simultaneously strengthen the matrix. These elements were introduced into the chemical composition of the Ni-based superalloy EP741NP produced by powder metallurgy. This led to the development of an experimental Ni-based HRA (NGK-6) of the following chemical composition in wt. %: (8–10) W, (4–5) Cr, (11–13) Co, (3–5) Mo, (2–2.5) Ti, (4.5–5) Al—[(1.5–2) Nb, (1–2) Ta, 0.5 Hf, 0.01 Zr] (package of low-alloy additions is given in square brackets) [12,44].

**Figure 9:**
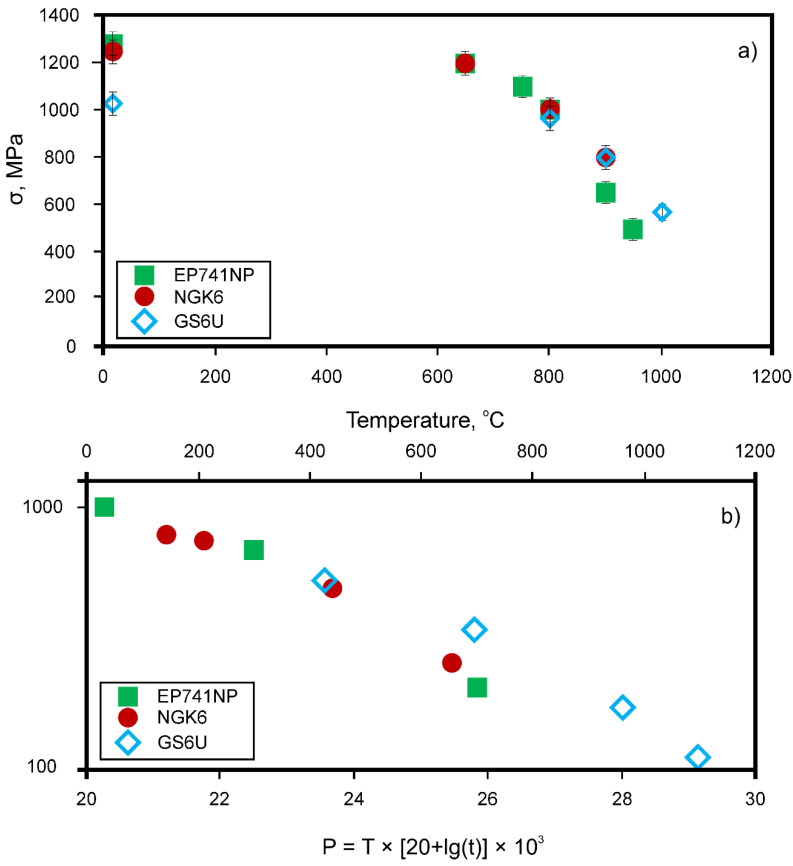
Mechanical testing of the Ni-based superalloys: tensile strength as a function of temperature (**a**), and the long-term strength as a Larson-Miller diagram (*P* = *T* [20 + log(*t*)] × 10^3^) (**b**); powder NGK-6 (circles), cast GS6U (triangles) and powder EP741NP (diamonds) [12]. Copyright 2015 Elsevier.

Figure 9 shows the results of testing the mechanical properties of the experimental NGK-6 alloy, as well as the typical cast superalloy GS6U (type MAR-M200) and powder (granular) superalloy EP741NP (for comparison). As expected, at room temperature (r.t.), the σ_b_ values of both powder alloys significantly exceed the σ_b_ of the cast alloy, although they are close to each other, Figure 9a. However, this difference decreases with increasing temperature; note that at a temperature of 900 °C, the strength of the experimental powder alloy NGK-6 significantly exceeds the strength of the powder alloy EP741NP (by about 150 MPa).

The indicators of the long-term strength of the alloys are shown in Figure 9b in the form of a Larson-Miller diagram (*P* = *T* [20 + log(*t*)] × 10^3^). The diagram shows that at high temperatures *T* and long life *t* (large values of the P parameter), the experimental NGK-6 powder alloy tends to have better creep resistance than the well-known EP741NP powder alloy.

## 6. Features of the Microstructure of Ni-Based Superalloys Obtained Using Additive Technologies

In the field of powder metallurgy, one of the most promising technologies for producing products from structural alloy powders is additive technologies (AT), which allows synthesizing complex-shaped objects of particular interest for aerospace engineering [45,46].

**Figure 10:**
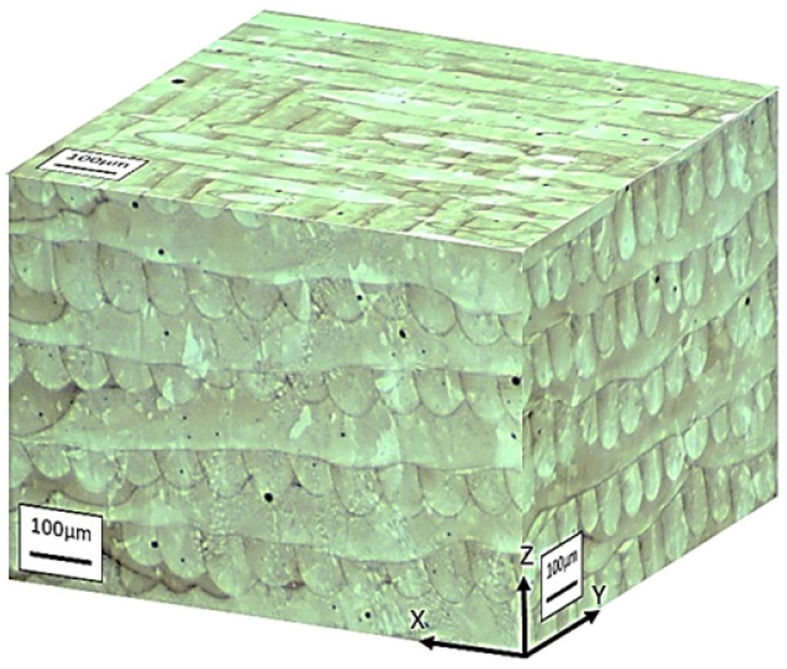
Optical metallographic image of Ni-base superalloy Inconel 718 obtained with SLM in as-built condition. The build direction is indicated by Z axis [46]. Copyright 2018 Elsevier.

A microstructure of additive object obtained by selective laser melting (SLM) from Ni-based superalloy Inconel 718, is shown in Figure 10 [46].

In the initial (as-built) state, as shown in Figure 10, a layered microstructure is observed, the characteristic defects of which are various types of internal interfaces (high-and low-angle grain boundaries, phase boundaries). The observed structural defects are, a priori, weak elements in terms of creep resistance. To increase the cohesive strength of defects, it is advisable to use surface-active transition metals as small additives that enrich and strengthen the interfaces. The introduction of small additives into alloys, apparently, can be carried out at the stage of powder production using the technology used to produce the NGK-6 powder alloy [44].

Another type of defect in the structure of additive objects are micropores, the total content of which can reach 1.35%, and they lead to a significant decrease in strength characteristics. All pores can be divided into two groups: (1) gas-filled pores (usually with Ar), and (2) gas-free pores. This separation is important because only the pores without gas could be perfectly closed by a HIP, and the use of HIP provides significant densification of the microstructure of additive objects and improvement of mechanical properties [47].

Such a positive effect of HIP is observed in the treating of many nickel HRAs [48,49]; Table 3 presents data for Ni-based superalloy EP741NP obtained by the SLM [49].

When HIPing additive objects made of nickel HRAs, it should be borne in mind that the formation of an optimal γ-γ′—microstructure usually does not occur during the HIP process, for this, a complete heat treatment must be carried out after the HIP, what ensures obtaining the required level of mechanical properties, Table 3.

Alloying elements and their influence on the cohesive properties of alloys are considered in the [50,51]; there are also some examples of the choice of alloying elements for chemical composition of alloys in accordance with the predictions of the theory.

## 7. Other Examples Application of the Approach: Beyond Ni-Based Alloys

Alloying of structural alloys with elements that increase cohesive strength and improve performance is used for design not only nickel HRAs, but also other metal alloys [52,53]. It is particularly necessary to emphasize the universal nature of the critical influence of refractory metals on the state of GBs in alloys with various structures (fcc (Ni), body-centered cubic (Cr), hexagonal close-packed (Ti)), which tend to brittle fracture at r.t. In all structures the most “useful” alloying elements should simultaneously increase the cohesive strength of the matrix phase (*E_coh_*) and the GB (*η*).

The effect of refractory metals on the cohesive properties of two matrix phases simultaneously with fcc (Ni) and bcc (Cr) lattices are investigated in [52]. It was established that Ta, Mo, Re and W can be used as effective strengtheners in chromium-based alloys, and Nb, Ir, and Ru effect is multidirectional: these elements increase GB’s cohesive strength but slightly soften the bcc matrix. Since Nb and Ta are both effective GB strengtheners in Ni, Figure 8, the authors [52] chose these elements as “useful” small additions in Cr-base alloys with two-phase fcc(Ni) and bcc(Cr) matrix.

Increasing the cohesive strength of alloys by alloying is particularly important for the development of a new generation of HRAs based on many refractory metals with melting temperatures above 2000 °C and potential operating temperatures of 1500 °C [54,55,56,57]. For instance, brittle fracture along GBs was shown in a high-temperature W-Ta–Mo–Nb alloy with a high alloying elements content [58]. The theoretical bcc metals tendency analysis toward GB brittle fracture [59] occurred that, among bcc metals, W and Mo are characterized via a high tendency toward GB fracture because of GB and external surface specific energies ratio, which determines the cohesive GBs strength. The segregation of the impurity at interfaces can change the ratio increases the plasticity in refractory metals. According to [60,61], alloying of special refractory additives also can be used to strengthen GBs in W, Mo, and their alloys.

## 8. Conclusions

In this review is analyzed the principles of alloying HRAs aimed at improving performance characteristics by increasing cohesive properties. The following are the important findings in the current review:

1. To select the chemical composition of HRAs, it is proposed to use an approach according to which the creep-rupture characteristics of alloys with a given microstructure at high temperatures are mainly determined by the cohesive properties of atoms in the matrix and at the grain boundaries, the parameters of which can be determined theoretically by using computer modeling based on the density functional theory. Using this approach, a series of nickel HRAs with mono- and polycrystalline structures was developed.

2. The effectiveness of this approach for the development of chromium- and titanium-based alloys is also shown. Increasing the cohesive strength of alloys by alloying is particularly important for the development of a new generation of HRAs based on many refractory metals.

3. To assess the structural stability of HRAs at elevated temperatures, models of diffusion coarsening of both “classical” structures with isolated particles of strengthening phase in the matrix and composite structures can be used.

## Figures and Tables

**Table 1:** Chemical compositions of single-crystal superalloys KS-(1-3) (wt. %).

Ni-Based Alloy	W	Ta	Re	Cr + Co + Ti + Nb + Al	Others, Nominal
KS-1	16.0	6.3	0	12.3	0.02La + 02Y + 0.02Ce
KS-2	15.0	6.3	2.0	13.3	0.02La + 02Y + 0.02Ce
KS-3	9.9	7.6	5.5	9.7	0.02La + 02Y + 0.02Ce

**Table 2:** The long-term strength of the fully treated KS-(1-3) alloys at the temperature 1000 °C.

Ni-Base Alloy	Chemical Alloy Composition (wt.%)	σ_100_^1000^ (MPa)	σ_500_^1000^ (MPa)
KS-1	16 W, 5Ta	255	194
KS-2	(12–15) W, 8Ta, 2Re	270	-
KS-3	10 W, 8Ta, 6Re	330	263
CMSX-2	8 W, 6Ta	226	180
CMSX-4	6 W, 7Ta, 3Re	270	190
CMSX-10M	5 W, 8Ta, 6Re	300	215
EPM-102	6 W, 8.25Ta, 6Re, 3Ru	330	240

**Table 3:** Changing the mechanical properties of the Ni-based superalloy EP741NP obtained by the SLM during subsequent HIP and complete heat treatment.

Sample Status	Ultimate Tensile Strength UTS (σ_B_) (MPa)	Yield Strength (σ_0,2_) (MPa)	Elongation δ (%)
As-built	1083 ± 27	853 ± 16	10.5 ± 4.3
After HIP	1292 ± 25	811± 5	24.9 ± 1.3
After HIP + complete heat treatment	1455 ± 52	1023 ± 34	21.4 ± 6.7
Standard P/M	1275	834	13

## Data Availability

Not applicable.
